# Intraoperative Ultrasound: A Tool to Support Tissue-Sparing Curative Pancreatic Resection in Focal Congenital Hyperinsulinism

**DOI:** 10.3389/fendo.2018.00478

**Published:** 2018-08-22

**Authors:** Julie Bendix, Mette G. Laursen, Michael B. Mortensen, Maria Melikian, Evgenia Globa, Sönke Detlefsen, Lars Rasmussen, Henrik Petersen, Klaus Brusgaard, Henrik T. Christesen

**Affiliations:** ^1^Department of Paediatrics, Odense University Hospital, Odense, Denmark; ^2^Department of Clinical Research, University of Southern Denmark, Odense, Denmark; ^3^Department of Surgery, Odense University Hospital, Odense, Denmark; ^4^OPAC, Odense Pancreas Centre, Odense University Hospital, Odense, Denmark; ^5^Department of Paediatric Endocrinology, Endocrine Research Centre, Moscow, Russia; ^6^Department of Paediatric Endocrinology, Ukrainian Centre of Endocrine Surgery, Kiev, Ukraine; ^7^Department of Pathology, Odense University Hospital, Odense, Denmark; ^8^Department of Nuclear Medicine, Odense University Hospital, Odense, Denmark; ^9^Department of Clinical Genetics, Odense University Hospital, Odense, Denmark

**Keywords:** congenital, hyperinsulinism, hypoglycemia, pancreas, surgery, ultrasound, histology, genetics

## Abstract

**Background:** Focal congenital hyperinsulinism (CHI) may be cured by resection of the focal, but often non-palpable, pancreatic lesion. The surgical challenge is to minimize removal of normal pancreatic tissue.

**Aim:** To evaluate the results of intraoperative ultrasound-guided, tissue-sparing pancreatic resection in CHI patients at an international expert center.

**Methods:** Retrospective study of CHI patients treated at Odense University Hospital, Denmark, between January 2010 and March 2017.

**Results:** Of 62 consecutive patients with persistent CHI, 24 (39%) had focal CHI by histology after surgery. All patients had a paternal *ABCC8* or *KCNJ11* mutation and a focal lesion by ^18^F-DOPA-PET/CT. Intraoperative ultrasound localized the focal lesion in 16/20 patients (sensitivity 0.80), including one ectopic lesion in the duodenal wall. Intraoperative ultrasound showed no focal lesion in 11/11 patients with diffuse CH (specificity 1.0). The positive predictive value for focal histology was 1.0, negative predictive value 0.73.

Tissue-sparing pancreatic resection (focal lesion enucleation, local resection of tail or uncinate process) was performed in 67% (*n* = 16). In 11/12 having tissue-sparing resection and intraoperative ultrasound, the location of the focal lesion was exactly identified. Eight patients had resection of the pancreatic head or head/body, four with Roux-en-Y, three with pancreatico-gastrostomy and one without reconstruction. None had severe complications to surgery. Cure of hypoglycaemia was seen in all patients after one (*n* = 21) or two (*n* = 3) pancreatic resections.

**Conclusion:** In focal CHI, tissue-sparing pancreatic resection was possible in 67%. Intraoperative ultrasound was a helpful supplement to the mandatory use of genetics, preoperative ^18^F-DOPA-PET/CT and intraoperative frozen sections.

## Introduction

Congenital hyperinsulinism (CHI) is the most common cause of persistent hypoglycaemia in infants with an incidence of 1–1.4 per 50,000 live births in populations without founder mutations (1). CHI is characterized by an unregulated hypersecretion of insulin from pancreatic beta cells despite hypoglycaemia (2, 3). A prompt and effective treatment is necessary to avoid neurological complications (4).

CHI is a very heterogeneous disease with respect to genetic cause, histology and clinical presentation (5). To date, mutations in 11 different genes are known to cause CHI, most commonly in the beta cell K_ATP_-channel genes *ABCC8* and *KCNJ11* (6). The two major histological subtypes are focal and diffuse CHI. The genetic basis of focal CHI is a two-hit event with a germ line mutation in *ABCC8* or *KCNJ11* on the paternal allele, combined with a focal somatic loss in pancreatic cells of the maternal chromosome 11p15 region including *ABCC8, KCNJ11* and the maternally imprinted tumor suppressor gene p57 and hence focal loss of heterozygosity. This causes focal adenomatous hyperplasia of beta cells with unregulated insulin secretion due to loss of K_ATP_-channel function (7).

Histologically, the focal lesions preserve the normal lobular structure of the pancreas with confluent islets of Langerhans. The beta cells within the lesion are hyperactive, sometimes with enlarged cytoplasm. Loss of tumor suppressor p57 staining is characteristic within the lesion and the beta cells outside the focus are resting with a high storage of insulin, which sometimes can be appreciated morphologically by their smaller size (8, 9).

Until the discovery of focal CHI (10), the main treatment of severe CHI was subtotal (95–98%) pancreatectomy to minimize the risk of further brain damage from hypoglycaemia, but with a recognized risk of developing insulin-dependent diabetes (11). Thus, limited (i.e., tissue-sparing) pancreatic resection in patients with focal CHI would represent a huge step forward in management. Preoperative pancreatic venous sampling was previously used to roughly identify the localisation of a focal lesion (12). In non-palpable lesions, patients underwent piece-meal pancreatic resections where suspicious areas from the pancreas were submitted for frozen section until the focal CHI lesion was identified (13). From 2006, PET/CT scan imaging with ^18^F-fluoro-dihydroxyphenylalanine (^18^F-DOPA) has been used as a more precise and non-invasive method for preoperative identification and localisation of focal lesions (14). This has helped in planning the surgical procedures. However, the focal lesions are often only a few millimeters in diameter, irregular in shape, may send octopus-like tentacles into the surrounding pancreatic tissue, invisible and non-palpable during surgery, which challenges identification and tissue-sparing surgery. Furthermore, the surgeons find it challenging to find a focal lesion in a three-dimensional pancreas from a two-dimensional image. Focal lesions can be embedded within the pancreatic tissue and may not be easily visible or even palpable. The more recent introduction of intraoperative ultrasound (IOUS)-guided resection has shown promising results (15, 16).

In this preliminary study, we aimed to evaluate the results of the surgical treatment of children with focal CHI at an international CHI center after the introduction of IOUS.

## Methods

### Setting

In this observational study, we retrospectively evaluated hospital files of CHI patients admitted to the International Hyperinsulinism Centre, Hans Christian Andersen Children's Hospital, Odense University Hospital, Denmark, between January 1, 2010 and March 31, 2017. Contacting the referring medical doctors of foreign patients ensured follow-up data after discharge.

Genetic analyses were performed by denaturing high-performance liquid chromatography with subsequent Sanger sequencing of ABCC8 and KCNJ11 until 2012 (17). From 2013 onwards, next generation sequencing with confirmative Sanger sequencing of a broader panel of CHI-related genes was used as in other institutions (18). Mutation databases, allele frequency and *in silico* prediction software programs were performed to establish the pathogenicity of DNA variations found. Only known mutations in CHI patients or rare mutations predicted as pathogenic were accepted.

^18^F-DOPA-PET/CT scans were acquired on a GE Discovery PET/CT scanner (GE Medical System, Waukesha, WI, USA), and analyzed on a Dexus Advantage Workstation server 2.0 or 3.2. An ^18^F-DOPA-PET/CT scan suggesting focal CHI was followed by pancreatic surgery at our center facilities. From August 2010, IOUS was performed using a high frequency (3–15 MHz) hockey stick transducer (L53K, Hitachi, Denmark) to aid the localisation of the pancreatic lesions, with exclusion of patients with intraoperative obvious palpable/visible lesions localized as predicted by ^18^F DOPA-PET/CT. Patients with predicted diffuse CHI by ^18^F DOPA-PET/CT were also subjected to IOUS for control. All patients had open surgery.

Pancreatic tissue histology was assessed by intraoperative frozen section analysis using hematoxylin-eosin staining with the addition of immunohistochemistry for synaptophysin in difficult cases. After surgery, the tissue specimens were analyzed by microscopy of hematoxylin-eosin (H&E) staining of formalin fixed, paraffin embedded 4 μm thick sections; immunohistochemical staining using the BenchMark Ultra immunostainer (Ventana Medical Systems, Tucson, AZ) with the OptiView-DAB detection kit (Ventana Medical Systems, Tucson, AZ); nuclear counterstaining with the BenchMark Ultra instrument using Hematoxylin II (Ventana Medical Systems, Tucson, AZ) and coverslipping using a Tissue-Tek Film coverslipper (Sakura, Alphen aan den Rijn, The Netherlands). According to the internal protocol at the Department of Pathology, Odense University Hospital, almost all tissue samples had supplementary post-surgery immunohistochemical examination, which included synaptophysin, chromogranin A, insulin, glucagon, somatostatin, and the imprinted maternally expressed tumor suppressor p57, for confirmation of the diagnosis, also in cases where H&E stained slides showed the characteristic features of focal CHI.

Focal CHI was histologically defined as focal adenomatous hyperplasia of beta cells as described by others (8, 9).

### Patients

Inclusion criteria for the present study were a diagnosis of hyperinsulinaemic hypoglycaemia (fasting hypoglycaemia accompanied by inappropriately elevated plasma insulin). Exclusion criteria were transient disease (spontaneous remission in less than 1 month), age at onset ≥18 years and no pancreatic surgery at Hans Christian Andersen Children's Hospital, Odense University Hospital, Denmark.

Patients with non-focal CHI by histology were included regarding data on IOUS only. For the included patients with focal CHI, the following data were recorded: Gender, age at first symptoms, age at first recorded hypoglycaemia (0–2 days of age <2.5 mmol/L; from 3 days of age <3.2 mmol/L), maximally recorded glucose infusion rate (mg/kg/min), age at surgery, type of surgery, IOUS findings, final diagnosis at histology, second pancreatic resection (yes/no; type), admission days after surgery, surgery time (minutes), postoperative hyperglycaemia (blood glucose ≥7.0 mmol/l ≥24 h) or hypoglycaemia events (values), number of days in need of intravenous (IV) glucose after surgery, early and late postoperative complications including diabetes (fasting blood glucose ≥7 mmol/L) and malabsorption, age at follow-up, and follow-up time after surgery. Moreover, persistent feeding disorder and neurological impairment (psychomotor retardation, epilepsy, cerebral palsy, reduced vision, or hearing loss) were recorded.

The pancreatic resections were classified according to size and topography as larger (total/subtotal head resection or more than 1/3 of the pancreas resected), or tissue-sparing (enucleation or partial resection involving less than 1/3 of the pancreas). Moreover, the difficulty of the pancreatic resection was classified as moderate (enucleation or partial resection to the left or on the portal vein), or high (all resections to the right of the portal vein). Early and late postoperative complications (early: ≤2 weeks; late: >2 weeks) were graded according to Clavien-Dindo (19) and supplemented with an evaluation of delayed gastric emptying and pancreatic fistula according to the definitions issued by the International Study Group for Pancreatic Surgery (20). Cure of hypoglycaemia was defined as normal blood glucose on full enteral eating, no i.v. glucose infusion or medication related to hypoglycaemia at latest follow-up.

Eleven of the included patients had their cerebral outcome described in details elswhere (21), but without data related to surgery.

The study complied with the STROBE statement for observational studies (22).

### Statistics

Descriptive statistics included means (SD) for normally distributed data, medians (range) for non-parametrical data and percentages. Comparison of groups was performed using student T test, Fischer's exact test or Chi-square test. IBM SPSS Statistics 24.0 was used for statistical calculations. A two-tailed *p* < 0.05 was considered statistically significant; 0.05–0.10 trends.

### Ethics

All patients were treated in accordance with the principles of good clinical practice from The Declaration of Helsinki. The Local Ethics Committee, no 56193 and Danish Data Protection Agency, no. 16/41306, approved the study protocol. Informed written consent was obtained from parents.

## Results

### Patient characteristics

**Figure 1:**
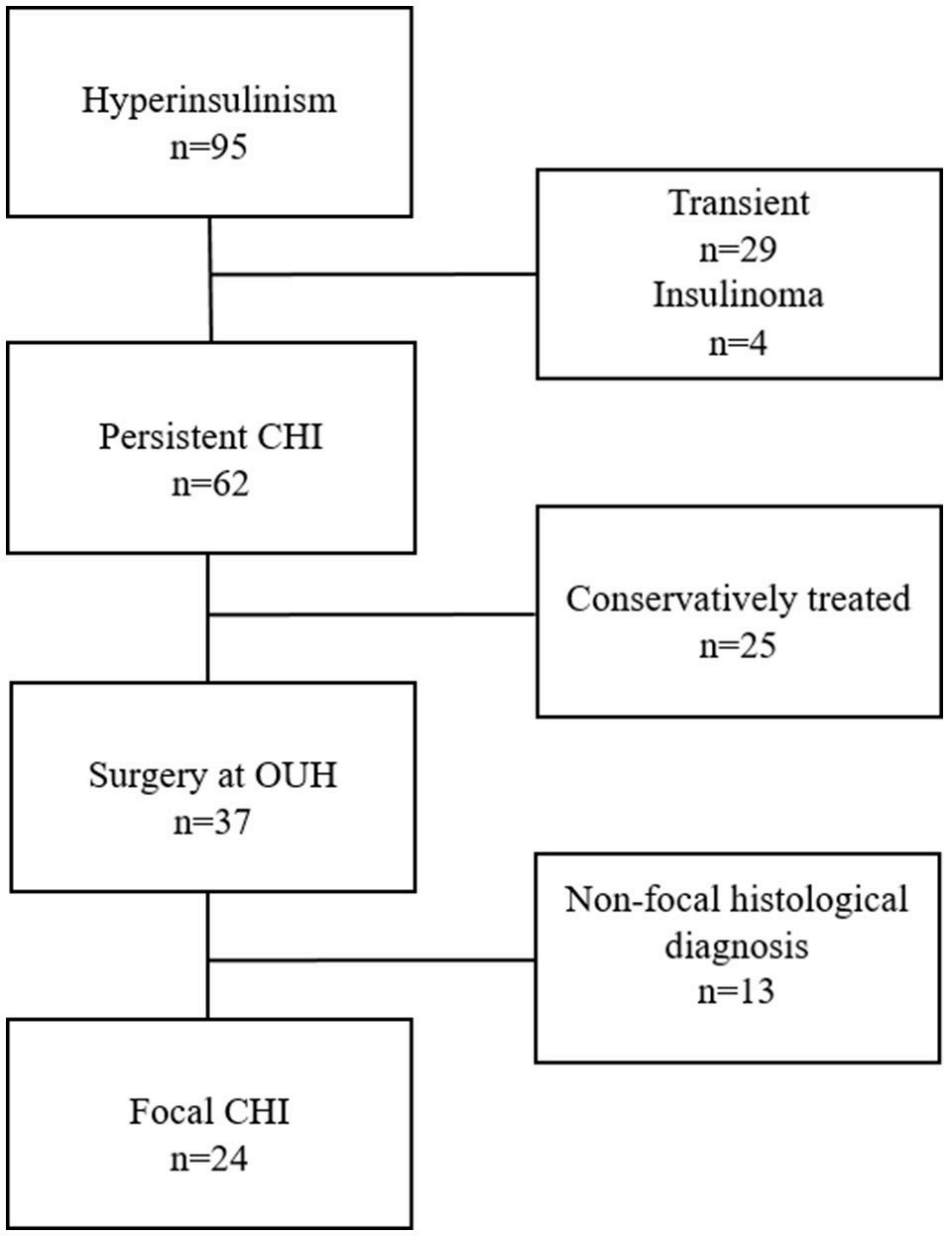
Patient inclusion flow chart.

Ninety-five patients were admitted to the Hans Christian Andersen Children's Hospital with a diagnosis of hyperinsulinism during the study period (Figure 1). Sixty-two patients were diagnosed with persistent CHI, of which 25 patients were excluded for being conservatively treated. None of these patients had a diagnosis of focal CHI by ^18^F-DOPA-PET/CT. Of the 37 surgically treated patients, 13 had a non-focal (diffuse or atypical) histological diagnosis, rendering 24 resected patients with focal CHI. The prevalence of focal CHI was 65% among all subjected to surgery; or 39% of all with persistent CHI.

Of the 24 patients with focal CHI, three patients were from Denmark. The other patients were referred from Russia (*n* = 10), Ukraine (*n* = 5), and Belarus, Latvia, Georgia, Kazakhstan, Singapore, and Sweden (*n* = 1 for all). Basal patient characteristics are resumed in Table 1.

**Table 1:** Characteristics of the 24 patients with focal CHI.

**GENDER**, ***n*** **(%)**	
Female	15 (63%)
Male	9 (37%)
Birth weight (grams), mean (SD), *n* = 20	3626 (600)
Gestational age (week), median (range), *n* = 20	40 (33-41)
**APGAR-SCORE AT 5 MIN**, ***n*** **(%)**, ***n*** = **12**	
Score (≥8)	8 (33%)
Score (≤7)	4 (17%)
Age at first symptoms (days), median (range), *n* = 23	2 (0–30)
Age at first recorded hypoglycaemia (days), median (range), *n* = 23	2 (0–30)
Maximal glucose infusion rate (mg/kg/min), median (range), *n* = 21	12.1 (4.4–28.3)
**GENETIC MUTATION**, ***n*** **(%)**	
*ABCC8*	23 (96%)
*KCNJ11*	1 (4%)
^**18**^**F-DOPA-PET/CT LOCALIZATION**, ***n*** **(%)**	
Head	11 (45.8%)
Body	4 (16.7%)
Tail	5 (20.8%)
Uncinate process	4 (16.7%)
Follow-up time after surgery (months), median (range)	12.5 (0.2–78.9)
Age at last follow-up (years), median (range)	1.7 (0.24–6.97)

All patients with focal CHI had a heterozygous paternal K_ATP_-channel mutation, *ABCC8*; *n* = 23 (96%), *KCNJ11*; *n* = 1 (Table 2). Five patients had novel missense mutations. Four unrelated patients had the same mutation, *ABCC8* p.Gln444His, which we frequently have found in Russian and East European CHI patients.

**Table 2:** Genetics of the 24 focal CHI patients.

**Patient no**.	**Heterozygous paternal K_ATP_-channel mutations**	**Country of origin**	**Mutation characteristics**
1	*ABCC8* c.2140C>T, p.Gln714*	Denmark	Truncating, novel
2	*ABCC8* c.1332G>T, p.Gln444His	Russia	Missense, prevalent
3	*ABCC8* c.2866del, p.Ser956FS*86	Russia	Truncating, novel
4	*ABCC8* c.1332G>T, p.Gln444His	Ukraine	Missense, prevalent
5	*ABCC8* c.4415-13G>A	Ukraine	Intronic
6	*ABCC8* c.1332G>T, p.Gln444His	Latvia	Missense, prevalent
7	*ABCC8* c.4309C>T, p.Arg1437*	Ukraine	Truncating
8	*ABCC8* c.4414G>A, p.Asp1472Asn	Russia	Missense
9	*ABCC8* c.415delC, p.Leu139fs*5	Denmark	Truncating, novel
10	*ABCC8* c.2921-2A>G	Sweden	Splice site
11	*ABCC8* c.580-1G>C	Denmark	Splice site
12	*ABCC8* c.3989-9G>A	Russia	Intronic, Ashkenazi origin
13	*ABCC8* c.4575+1G>C	Russia	Splice site novel
14	*ABCC8* c.2506C>T, p.Arg836*	Kazakhstan	Truncating, novel
15	*KCNJ11* c.998T>C, p.Phe333Ser	Ukraine	Missense
16	*ABCC8* c.2767C>T, p.Gln923*	Ukraine	Truncating
17	*ABCC8* c.1332G>T, p.Gln444His	Russia	Missense, prevalent
18	*ABCC8* c.4059C>A, p.Tyr1353*	Belarus	Truncating
19	*ABCC8* c.3557+1G>A	Russia	Splice site
20	*ABCC8* c.4238C>T, p.Pro1413Leu	Russia	Missense
21	*ABCC8* c.4198+1G>C	Georgia	Splice site
22	*ABCC8* c.4477C>T, p.Arg1493Trp	Russia	Missense
23	*ABCC8* c.4477C>T, p.Arg1493Trp	Singapore	Missense
24	*ABCC8* c.1032C>G, p.Tyr344*	Russia	Truncating

### Surgery

**Figure 2:**
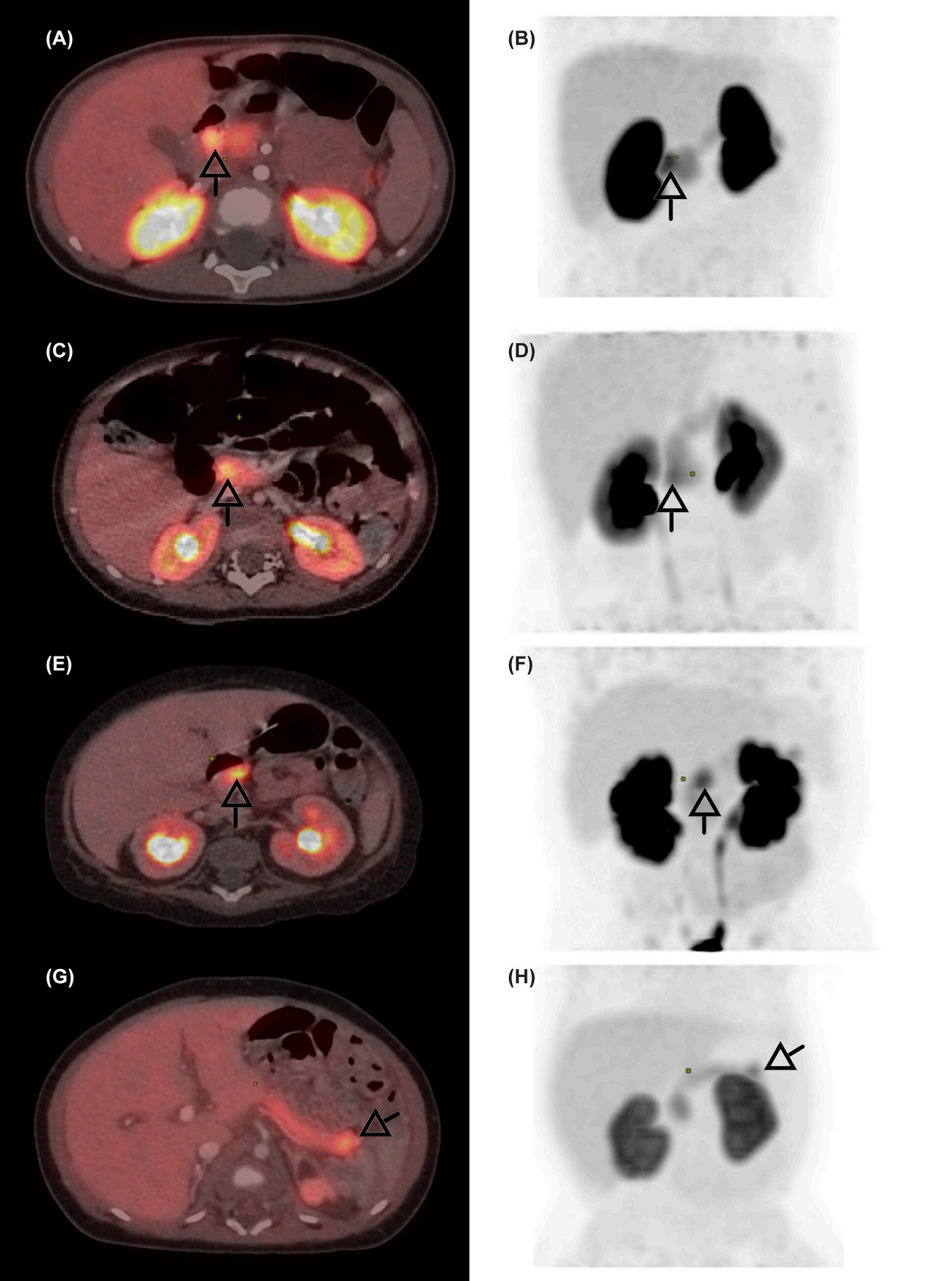
^18^F-DOPA-PET/CT scans of four patients with focal CHI. The focal lesions are seen in the duodenum **(A,B)**, the pancreatic head **(C,D)**, body **(E,F)**, and tail **(G,H)**. Arrows indicate the focal lesion.

**Figure 3:**
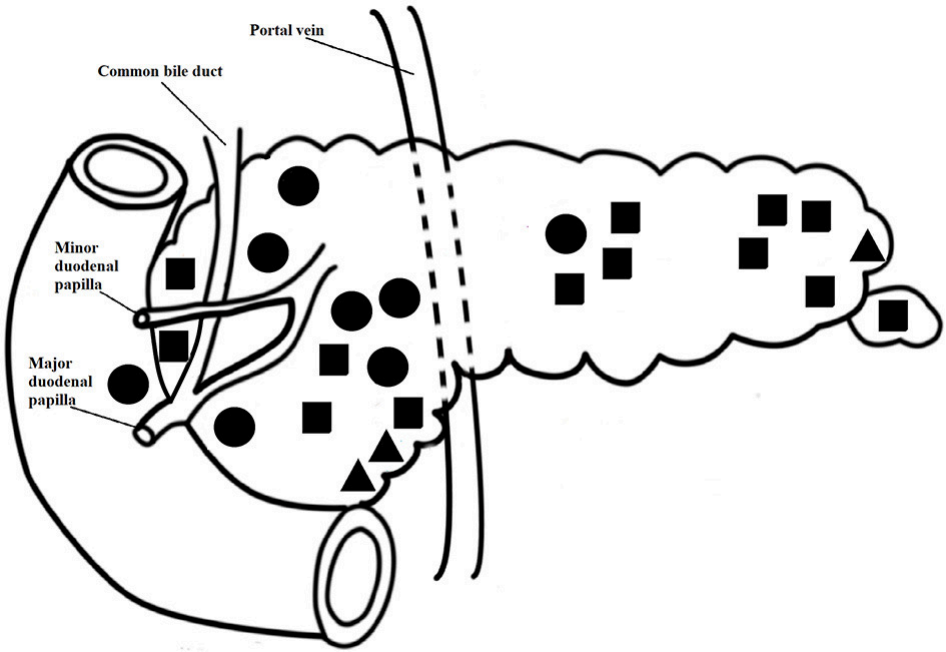
Localisation of the focal lesions in the pancreas and duodenum and type of surgery. The dots do not depict the lesion size. Square: enucleation; triangle: resection of the tail or uncinate process; solid circle: large resection.

**Figure 4:**
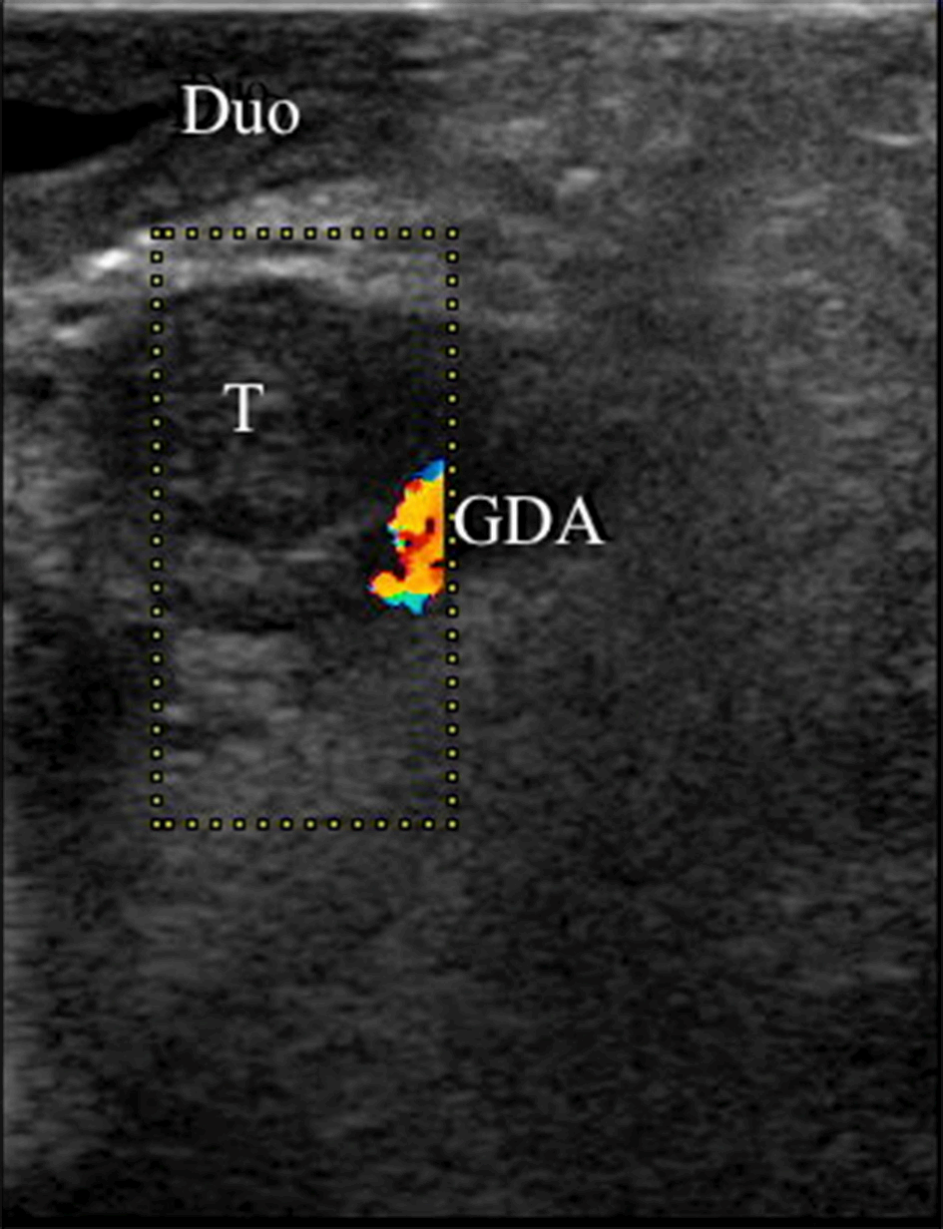
Intraoperative ultrasound of a focal CHI lesion. A nine mm hypo-echoic focal CHI lesion (“T”) is identified adjacent to the gastroduodenal artery (“GDA”) and the duodenum (“Duo”).

All patients had only one focal lesion and all focal lesions (100%) were correctly identified and roughly localized by ^18^F-DOPA-PET/CT scan (Figures 2, 3). The median (range) age at surgery was 4.8 (0.8–28.9) months (Table 3). IOUS was used together with frozen section in 20 patients (83%), (Figure 4). In the last four patients, the lesion was identified visually and/or by palpation.

**Table 3:** Pancreatic surgery details in the 24 patients with focal CHI.

		***p*-value**
Age at surgery, months, median (range)	4.8 (0.8–28.9)	
Intraoperative ultrasound, *n* (%)	20 (83%)	
Hospitalization days after surgery, median (range)	10 (3–84)	
Size of lesion by histology, millimeters, median (range)	8 (3–35)	
Tissue sparing resection, *n* (%)	16 (67%)	
Enucleation, *n* (%)	13 (81%)	
Resection of tail or uncinate process, *n* (%)	3 (19%)	
Large resection, *n* (%)	8 (33%)	
Roux-en-Y pancreatico-jejunostomy reconstruction	4 (50%)	
Pancreatico-gastrostomy reconstruction	3 (37.5%)	
No reconstruction	1 (12.5%)	
Difficulty of resection		
Left - or on the portal vein, *n* (%)	10 (42%)	
Right of the portal vein, *n* (%)	14 (58%)	
Surgery time, minutes, median (range), *n* = 22	105 (40–255)	
Left vs. right of portal vein, median minutes	92 vs. 139	0.02
Tissue sparing vs. large resection, median minutes	102 vs. 158	0.14
Patients with hypoglycaemia events after surgery, *n* (%)	8 (33%)	
Lowest blood glucose after surgery, mmol/L, mean	2.2 (0.8)	
(SD)		
I.V. glucose for hypoglycaemia after surgery, days,	1 (0–8)	
median (range)		
Cured after 1st pancreatic operation, *n* (%)	21 (87.5%)	
Cured after 2nd pancreatic operation, *n* (%)	3 (12.5%)	

The IOUS identified the focal lesion correctly in 16/20 patients. In one patient, no lesion was identified by ultrasound; in three the result was uncertain. IOUS identified non-focal disease in 11/11 patients with diffuse histology; two diffuse patients had no ultrasound performed. The four patients with failure of identifying the focal lesion by IOUS either had a large pancreatic head lesion (*n* = 3), or a smaller lesion in the uncinate process (*n* = 1). Accordingly, IOUS had a sensitivity of 0.80, a specificity of 1.00, a positive predictive value of 1.0 and a negative predictive value of 0.73.

Sixteen (67%) patients had tissue-sparing pancreatic resections. Of these, 13 patients had focal lesion enucleation with resection of the lesion itself and less than 5% of the surrounding normal pancreatic tissue (body or tail; *n* = 8, pancreatic head; *n* = 4, uncinate process; *n* = 1). The latter three patients had a partial tail resection. The tissue-sparing resections were aided by ^18^F-DOPA PET/CT and IOUS in 11; by ^18^F-DOPA PET/CT and intraoperative palpation and/or vision in four; and by ^18^F-DOPA PET/CT with uncertain IOUS in one.

**Figure 5:**
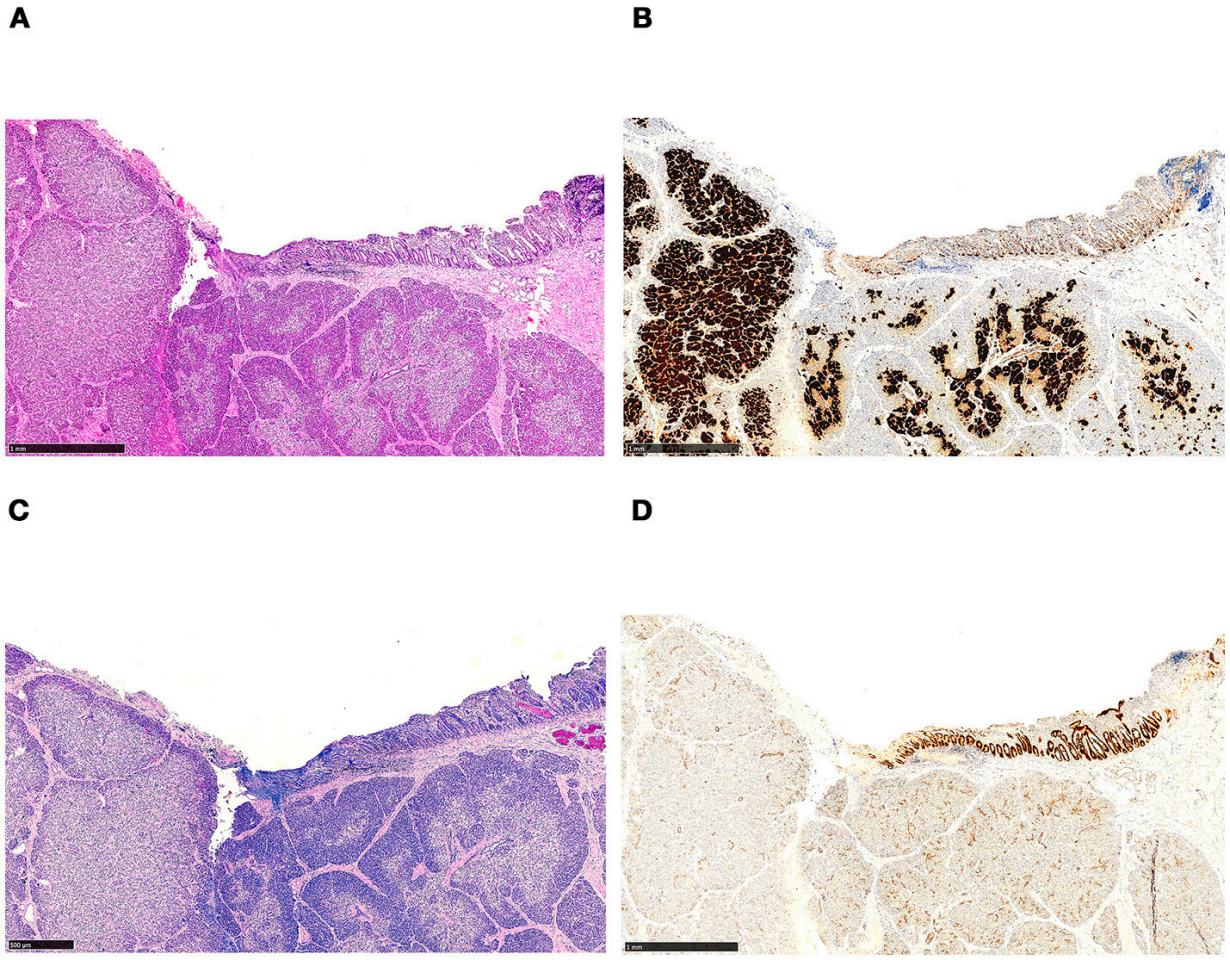
Histology of ectopic pancreatic tissue located in the wall of the duodenum, revealing features of focal CHI. The tissue section has been used for intraoperative frozen section analysis prior to fixation and paraffin embedding. **(A)** Focal adenomatous hyperplasia, showing endocrine cells involving more than 40% of the area (H&E staining). **(B)** The endocrine nature of the cells is emphasized by immunohistochemistry (synaptophysin immunostaining). **(C)** In the upper right corner, the duodenal surface with mucosa containing a few Brunner's glands is shown (Alcian blue periodic acid-Schiff staining). **(D)** Strong expression of CDX-2 in the duodenal mucosa (CDX2 immunostaining).

Eight patients (33%) had larger resections of the pancreatic head (*n* = 6), or head/body (*n* = 2). Reconstruction was performed in seven patients (Roux-en-Y pancreatico-jejunostomy, *n* = 4, pancreatico-gastrostomy, *n* = 3). One patient had an ectopic focal lesion, identified by ^18^F-DOPA-PET/CT to be located in the pancreatic head and/or in the adjacent duodenal wall. The lesion was neither palpable nor visible, but IOUS together with frozen section analysis confirmed an eight-mm lesion to be located in ectopic pancreatic tissue in the duodenal wall, ~5 mm from the major duodenal papilla (Figures 2A,B, 5). The lesion was removed with a subtotal pancreatic head resection combined with a small duodenotomy. Final histology confirmed the lesion to be located in ectopic pancreatic tissue in the duodenal wall and extending into the head of pancreas.

Three patients had a pancreatico-gastrostomy reconstruction after resection of the pancreatic head (*n* = 2) or head/body (*n* = 1). In two of these patients, IOUS could not localize the focal lesion. In both patients, the pancreatic head felt firmer than the rest of the pancreas, but the lesions could not be visually identified. Guided by frozen section, a focal lesion was found intraoperatively in both patients (total number of frozen sections in these patients: six and two, respectively). Both focal lesions were relatively large, measuring 20 mm and 35 mm in greatest dimension. Both patients received a resection of the pancreatic head and part of the body. The lesion of the third patient was located to the pancreatic head by IOUS, confirmed by frozen section, and enucleated. During the enucleation, the pancreatic duct was injured with leakage of pancreatic juice and a pancreatico-gastrostomy reconstruction was performed.

Three patients had a second surgery (large pancreatic resection with Roux-en-Y reconstruction; *n* = 2, partial pancreatic body and tail resection; *n* = 1) after a first attempt to perform focal lesion enucleation 1–13 days prior, but with involvement of resection margins at histology and relapse of persistent hypoglycaemia. All patients were cured after the second operation.

The difficulty of the pancreatic resection was classified as moderate (to the left or on the portal vein) in 10 patients (42%) and high (right of the portal vein) in 14 patients (58%).

The median (range) surgery time from incision to closure was 105 (40–255) min. Large pancreatic resections lasted on an average of 158 vs. 102 min for tissue sparing resections, *p* = 0.138. The operating time was significantly longer for resections classified as high difficulty vs. moderate difficulty, 139 vs. 92 min, *p* = 0.020. The median (range) hospitalization time after surgery was 10 (3–84) days.

By histology, the focal lesions had a median (range) diameter of eight (3–35) mm. The median number of frozen biopsies preceding the resection per patient was two (range 1–7). In 56 out of 57 (98%) specimens, the frozen section analysis was consistent with the final histological diagnosis. In the one exception, the frozen section analysis was “probably diffuse CHI” in the first biopsy, after which the lesion was enucleated during the same operation, however without further frozen sections. On post-surgery analysis after formalin fixation and paraffin embedding, the final histological analysis of the first biopsy showed normal pancreatic tissue and the enucleated tissue showed focal CHI.

### Early postoperative complications

Three (12%) patients had early postoperative complications. One patient had postoperative bleeding from the stomach, which did not need treatment (Clavien-Dindo I), one had a grade A pancreatic fistula with a maximal drain amylase concentration of 1145 U/L (Clavien-Dindo I), and one had delayed gastric emptying which was treated with an endoscopically placed duodenal feeding tube (Clavien-Dindo IIIb).

Patients with tissue-sparing pancreatic resections had less early postoperative complications compared to patients with larger resections (0/16 vs. 3/8, *p* = 0.028). There was no difference in early postoperative complication rates when comparing moderate with high difficulty pancreatic resections (0/10 vs. 3/14, *p* = 0.24).

### Late postoperative complications

The median (range) follow up time after surgery was 12.5 (0.2–78.9) months. Late postoperative complications occurred in two patients (8%); one had an incisional hernia (Clavien-Dindo IIIb) 4 months after discharge provoked by a severe attack of epilepsy, and one patient had small bowel obstruction from adherences (Clavien-Dindo IIIb). Both patients were surgically treated in their country of origin. No patients had reports of diabetes or malabsorption.

### Neurology and other outcomes

At last follow-up by age 1.7 (range 0.24–6.97) years, 12 (50%) had neurological impairment, especially psychomotor retardation (*n* = 12), Table 4. There was no correlation between a low Apgar-score (*p* = 1.00), age at disease onset (*p* = 0.537), or data related to surgery and neurological impairment. Surprisingly, a significantly *lower* mean (SD) maximally recorded glucose infusion rate was seen in patients with, compared to patients without, neurological impairment, 9.5 (3.4) vs. 15.5 (6.2) mg/kg/min, *p* = 0.016.

**Table 4:** Neurological impairment and other outcomes of the 24 focal CHI patients.

	***n* (%)**
Neurological complications, total	12 (50)
Psychomotor retardation	12 (50)
Epilepsy, *n* = 23	5 (21)
Reduced vision	2 (8)
Cerebral palsy, *n* = 22	3 (13)

Feeding disorder was seen in two (8%) of the patients, both with psychomotor retardation. At last follow-up (age 7.8 months, and 24.2 months, respectively), one had a duodenal feeding tube and the other a gastrostomy feeding tube.

## Discussion

This is the first report on a cohort of CHI patients in details describing the use of IOUS and clinical context supporting its use. All 24 patients were surgically treated with resultant cure of hypoglycaemia. IOUS had a sensitivity of 0.80 and a specificity of 1.0 in precisely localizing the focal lesion for the surgeon. In 11/12 (92%), IOUS led to tissue-sparing pancreatic resection with enucleation or partial resection of the tail or uncinate process. Another four had tissue-sparing resections after visual inspection and/or palpation only. Together, this led to tissue-sparing resections in 16 (67%) of the patients. The early complication rate was low and significantly lower after tissue-sparing pancreatic resection than after larger resections.

Our prevalence proportions of focal CHI, 39% of all with persistent CHI and 65% of surgery-treated patients, were comparable to other centers (5, 23, 24). Alike other centers, our prevalences were not population-based, as many patients were selected to referral for suspected focal CHI after genotyping. We saw a predominance of girls (63%) as also found by some (25, 26), but not all (27), suggesting chance findings. The median size of the focal lesions of eight mm and a maximum of 35 mm were comparable to other studies (15, 28).

The heterozygous, paternal K_ATP_-channel mutations identified were predominantly *ABCC8* mutations as in other studies (27, 29). Five patients had novel mutations at the time of investigation. Four unrelated East-European or Russian patients had an *ABCC8* mutation p.Gln444His, which we also have frequently found in other CHI patients in these populations suggesting a founder mutation.

The aid of IOUS to perform tissue-sparing pancreatic resection in 11 patients represents an important improvement in focal CHI surgery. In older studies, prior to the use of ^18^F-DOPA PET/CT, subtotal or near-total pancreatectomy was predominantly used in the treatment of focal CHI (30, 31), despite the high risk of diabetes and malabsorption after near-total pancreatectomy (32). A French center (26) reported in 2002 of 45 children with focal CHI with more extensive pancreatic resections, a higher number of complications, and a cure rate of 98%, one still being hypoglycaemic after four surgeries. In 2004, the same center (33) reported on 59 infants with focal CHI, treated with partial pancreatectomy of the tail/body, head or isthmus based on intraoperative identification by the surgeon and/or multiple biopsies with frozen sections. The Philadelphia center (13) reported in 2004 of 38 focal CHI patients treated with partial pancreatectomy. The extent of the pancreatectomies ranged from 5 to 98%, with 37% having more than 50% of the pancreas resected. Three patients required redo surgery due to hypoglycaemia, of whom two were cured, making up a cure rate of 92%. The same center reported in 2013 (1) on 138 children surgically treated for focal CHI, guided by ^18^F-DOPA PET/CT in the majority of patients, and intraoperative multiple biopsies and frozen sections. Of the focal lesions in the pancreatic head, 39% (23/59) were treated with near-total head resection or a Whipple procedure. In a study reviewing patients from 1980-2008 (the majority had no ^18^F-DOPA PET/CT), an average of 50% the pancreatic tissue was resected in 54 patients with focal CHI (34). In a recent German study with use of ^18^F-DOPA PET/CT including 30 patients with focal CHI, an estimated average of 20% of the pancreas was resected, 20% had Roux-en-Y reconstruction, and the cure rate after one (*n* = 27), two (*n* = 2) or three (*n* = 1) surgeries was 93% (28/30) (27).

Although based on retrospective data, we believe that our extensive use of IOUS allowed tissue-sparing pancreatic resection with the use of fewer intraoperative frozen sections, with a comparable low reoperation rate and a hypoglycaemia cure rate of 100%. Moreover, our median surgery time was short, 105 min, compared to e.g., 228 min by laparoscopy and 406 min by open resections in a recent German study (27), probably mostly owing to avoidance of multiple biopsies. The relatively low sensitivity (0.80) of IOUS to detect the lesion was attributed to the learning process. Our surgery was in all patients guided by ^18^F-DOPA-PET/CT as in other recent publications (1, 27, 35). The ^18^F-DOPA-PET/CT does not depict the major pancreatic and the intra-pancreatic common bile ducts (15), and exact same positioning of the infant during ^18^F-DOPA-PET/CT and surgery is regarded crucial for localisation of the focal lesion during surgery (16). In our experience, IOUS allowed visualization of the ducts, relevant vessels (e.g., branches from gastroduodenal artery and portal vein) and localisation of the focal lesion in real time, thus making the positioning of the patients unimportant. IOUS as a second imaging after ^18^F-DOPA-PET/CT in focal CHI surgery has previously been described as promising (15, 28, 36). Our preliminary series document for the first time in a patient cohort the advantage of IOUS to minimize resection of healthy pancreatic tissue and shorten surgery time as multiple biopsies become redundant.

Some centers use laparoscopy for pancreatic tail resections in focal CHI, but with a much longer operating time compared with the present data. Moreover, the resection may not always be curative (27). Furthermore, the laparoscopic approach is dependent on preoperative identification of the focal lesion as predicted by heterozygous paternal K_ATP_-channel mutations and ^18^F-DOPA-PET/CT (29, 37, 38). The sensitivity and specificity of these diagnostic tools do, however, not reach 100% (1, 27, 39–41), neither in our series (data not shown).

We identified three patients (12%) with early surgery complications, of which only one was classified above Clavien-Dindo I. This patient had transient feeding problems with post-operative delayed gastric emptying. Transient feeding problems have been reported in many patients with CHI (42).

The rate of surgery-related early complications in CHI varies substantially (13, 30), and comparisons are hampered by studies reporting outcome from a mixture of focal and diffuse CHI. Secondly, the classification (e.g., severity) of complications is not always reported. Postoperative morbidity is higher in diffuse CHI due to the extent of resection, but even in focal CHI high complication rates has been reported (30). We encountered no severe early or late surgical complications, and the risk of complications was very low (12% early, 8% late) suggesting that surgery is safe in patients with focal CHI.

None of our patients developed malabsorption or diabetes, as frequently seen after near-total pancreatectomy (36). Such complications should not be expected after tissue-sparing resection, and we did not observe any of the kind even after larger pancreatic resections. This included the three patients with a pancreatico-gastrostomy reconstruction, however, with a relatively short follow-up time of 30, 45, and 46 months, respectively. This reconstruction procedure has to our knowledge never been reported for infants with focal CHI. One of these patients had a transient pancreatic fistula after the surgery, requiring no further treatment but pancreatic drain. In a controlled trial in adults, pancreatico-gastrostomy reconstructions had a lower rate of pancreatic fistula compared to pancreatico-jejunostomy reconstructions after pancreatico-duodenectomy (43). Pancreatico-gastrostomy reconstruction may be used as an alternative to Roux-en-Y reconstruction in infants with focal CHI with need of pancreatic head resection (13), although data are sparse and minimal pancreatic resection should be the preferred procedure if possible.

In one patient, we identified an ectopic focal lesion in the duodenal wall very close to the major duodenal papilla, extending into the pancreas. Ectopic focal CHI lesions have been described a few other times with need of repeat surgery up to three times before identification and cure (1, 23, 44). Our patient had only one surgery (pancreatic head resection and Roux-en-Y reconstruction) with four frozen biopsies, a surgery time of 220 min and no early or late complications, giving further support to the efficiency of a protocol with ^18^F-DOPA-PET/CT plus IOUS with few frozen sections and short surgery time, even for ectopic lesions.

Children with focal CHI are at high risk of neurodevelopmental impairment (4, 21, 34, 45). Early diagnosis and sufficient treatment are crucial to avoid this (21). As disease severity usually correlates with cerebral damage, we were surprised to find a significant, *inverse* association between the maximally recorded glucose infusion rate and neurological impairment. This finding is, however, in line with an Australian study (31), where conservatively treated children with later onset and milder disease more frequently had neurological deficits than those treated surgically with more severe disease and earlier onset, although all had become normoglycaemic 35 days after presentation. The less apparent symptoms of milder hypoglycaemia may cause delay in diagnosis and treatment and hence result in worse neurological outcome compared to those presenting with convulsions and loss of consciousness. Of note, our median disease onset age was 2 days, although one at the extreme presented on day 30, but had normal neurodevelopmental outcome.

No association between data related to surgery and neurodevelopmental impairment was found. We neither found associations to Apgar score or age at disease onset (symptoms or diagnosis of hypoglycaemia). Most likely, undertreatment of severe hypoglycaemia linked to delay in diagnosis and adequate management at, or in collaboration with, an expert CHI center was causative (21), but we did not have systematic data from the referring foreign hospitals to detect a such association in the present study. Feeding disorder persisted in two (8%), despite cure of hypoglycaemia at last follow-up, however with a relatively short follow-up time to 7.8 and 24.2 months' age. Both patients had neurodevelopmental impairment and the feeding disorder was not interpreted as surgery complications. Banerjee et al. (42) reported persistent feeding problems in 34% after 6 months. The feeding problems correlated with the disease severity, rather than medication, nasogastric tube or gastrostomy tube feeding. The nature of long-lasting or persistent feeding problems in CHI remains to be studied in further details, although cerebral damage as a consequence of prolonged, severe hypoglycaemia seems to be a prominent cause.

Limitations of our study included the retrospective nature with hospital file data from several centers and countries and the relatively short median follow-up time of 12.5 months. Strengths included the relatively large sample size for a rare disease and detailed data sampling from patient files.

## Conclusion

Tissue-sparing pancreatic resection of focal CHI was possible in 67% of the patients with few and mild complications and 100% cure rates for hypoglycaemia. Added on genetics, ^18^F-DOPA-PET/CT and intraoperative frozen biopsies, IOUS was a helpful tool to limit surgical resection of normal pancreatic tissue in infants with CHI.

## Author contributions

JB, ML, and HC collecting data, statistics, abstract, introduction, methods, results, discussion, conclusion, tables, and figures. MBM and SD collecting data, methods, results, discussion. MM and EG collecting data, discussion. LR collecting data, methods, discussion. HP and KB collecting data, methods.

### Conflict of interest statement

The authors declare that the research was conducted in the absence of any commercial or financial relationships that could be construed as a potential conflict of interest.

## References

[1] Laje P Stanley CA Palladino AA Becker SA Adzick NS. Pancreatic head resection and Roux-en-Y pancreaticojejunostomy for the treatment of the focal form of congenital hyperinsulinism. J Pediatr Surg. (2012) 47:130–5. 10.1016/j.jpedsurg.2011.10.032 22244405 PMC3595012

[2] Ismail D Kapoor RR Smith VV Ashworth M Blankenstein O Pierro A. The heterogeneity of focal forms of congenital hyperinsulinism. J Clin Endocrinol Metab. (2012) 97:E94–9. 10.1210/jc.2011-1628 22031516 PMC7611920

[3] Senniappan S Arya VB Hussain K. The molecular mechanisms, diagnosis and management of congenital hyperinsulinism. Indian J Endocrinol Metab. (2013) 17:19–30. 10.4103/2230-8210.107822 23776849 PMC3659902

[4] Menni F de Lonlay P Sevin C Touati G Peigne C Barbier V. Neurologic outcomes of 90 neonates and infants with persistent hyperinsulinemic hypoglycemia. Pediatrics (2001) 107:476–9. 10.1542/peds.107.3.476 11230585

[5] Lord K Dzata E Snider KE Gallagher PR De Leon DD. Clinical presentation and management of children with diffuse and focal hyperinsulinism: a review of 223 cases. J Clin Endocrinol Metab. (2013) 98:E1786–9. 10.1210/jc.2013-2094 24057290 PMC3816257

[6] Stanley CA. perspective on the genetics and diagnosis of congenital hyperinsulinism disorders. J Clin Endocrinol Metab. (2016) 101:815–26. 10.1210/jc.2015-3651 26908106 PMC4803157

[7] James C Kapoor RR Ismail D Hussain K. The genetic basis of congenital hyperinsulinism. J Med Genet. (2009) 46:289–99. 10.1136/jmg.2008.064337 19254908

[8] Suchi M MacMullen CM Thornton PS Adzick NS Ganguly A Ruchelli ED. Molecular and immunohistochemical analyses of the focal form of congenital hyperinsulinism. Mod Pathol. (2006) 19:122–9. 10.1038/modpathol.3800497 16357843

[9] Rahier J Guiot Y Sempoux C. Morphologic analysis of focal and diffuse forms of congenital hyperinsulinism. Semin Pediatr Surg. (2011) 20:3–12. 10.1053/j.sempedsurg.2010.10.010 21185997

[10] Goossens A Gepts W Saudubray JM Bonnefont JP Nihoul F Heitz PU. Diffuse and focal nesidioblastosis. A clinicopathological study of 24 patients with persistent neonatal hyperinsulinemic hypoglycemia. Am J Surg Pathol. (1989) 13:766–75. 10.1097/00000478-198909000-00006 2669541

[11] Shilyansky J Fisher S Cutz E Perlman K Filler RM. Is 95% pancreatectomy the procedure of choice for treatment of persistent hyperinsulinemic hypoglycemia of the neonate? J Pediatr Surg. (1997) 32:342–6. 10.1016/S0022-3468(97)90207-4 9044150

[12] Brunelle F Negre V Barth MO Fekete CN Czernichow P Saudubray JM. Pancreatic venous samplings in infants and children with primary hyperinsulinism. Pediatr Radiol. (1989) 19:100–3. 10.1007/BF02387895 2537942

[13] Adzick NS Thornton PS Stanley CA Kaye RD Ruchelli E. A multidisciplinary approach to the focal form of congenital hyperinsulinism leads to successful treatment by partial pancreatectomy. J Pediatr Surg. (2004) 39:270–5. 10.1016/j.jpedsurg.2003.11.019 15017536

[14] Otonkoski T Nanto-Salonen K Seppanen M Veijola R Huopio H Hussain K. Noninvasive diagnosis of focal hyperinsulinism of infancy with [18F]-DOPA positron emission tomography. Diabetes (2006) 55:13–8. 10.2337/diabetes.55.01.06.db05-1128 16380471

[15] von Rohden L Mohnike K Mau H Eberhard T Mohnike W Blankenstein O. Visualization of the focus in congenital hyperinsulinism by intraoperative sonography. Semin Pediatr Surg. (2011) 20:28–31. 10.1053/j.sempedsurg.2010.10.011 21186001

[16] von Rohden L Mohnike K Mau H Eberhard T Mohnike W Blankenstein O. Intraoperative sonography: a technique for localizing focal forms of congenital hyperinsulinism in the pancreas. Ultraschall in der Medizin (Stuttgart, Germany : 1980). (2011) 32:74–80. 10.1055/s-0029-1245598 21305438

[17] Christesen HB Brusgaard K Alm J Sjoblad S Hussain K Fenger C. Rapid genetic analysis in congenital hyperinsulinism. Hormone Res. (2007) 67:184–8. 10.1159/000097063 17114887

[18] Flanagan SE Xie W Caswell R Damhuis A Vianey-Saban C Akcay T. Next-generation sequencing reveals deep intronic cryptic ABCC8 and HADH splicing founder mutations causing hyperinsulinism by pseudoexon activation. Am J Hum Genet. (2013) 92:131–6. 10.1016/j.ajhg.2012.11.017 23273570 PMC3542457

[19] Clavien PA Barkun J de Oliveira ML Vauthey JN Dindo D Schulick RD. The Clavien-Dindo classification of surgical complications: five-year experience. Annal Surg. (2009) 250:187–96. 10.1097/SLA.0b013e3181b13ca2 19638912

[20] Bassi C Marchegiani G Dervenis C Sarr M Abu Hilal M Adham M. The 2016 update of the International Study Group (ISGPS) definition and grading of postoperative pancreatic fistula: 11 years after. Surgery (2017) 161:584–91. 10.1016/j.surg.2016.11.014 28040257

[21] Helleskov A Melikyan M Globa E Shcherderkina I Poertner F Larsen AM. Both low blood glucose and insufficient treatment confer risk of neurodevelopmental impairment in congenital hyperinsulinism: a multinational cohort study. Front Endocrinol. (2017) 8:156. 10.3389/fendo.2017.00156 28740482 PMC5502348

[22] von Elm E Altman DG Egger M Pocock SJ Gotzsche PC Vandenbroucke JP. The Strengthening the reporting of observational studies in epidemiology (STROBE) statement: guidelines for reporting observational studies. J Clin Epidemiol. (2008) 61:344–9. 10.1016/j.jclinepi.2007.11.008 18313558

[23] Hussain K Seppanen M Nanto-Salonen K Adzick NS Stanley CA Thornton P. The diagnosis of ectopic focal hyperinsulinism of infancy with [18F]-dopa positron emission tomography. J Clin Endocrinol Metab. (2006) 91:2839–42. 10.1210/jc.2006-0455 16684819

[24] de Lonlay P Fournet JC Touati G Groos MS Martin D Sevin C. Heterogeneity of persistent hyperinsulinaemic hypoglycaemia. A series of 175 cases. Eur J Pediatr. (2002) 161:37–48. 10.1007/s004310100847 11808879

[25] de Lonlay-Debeney P Poggi-Travert F Fournet JC Sempoux C Dionisi Vici C Brunelle F. Clinical features of 52 neonates with hyperinsulinism. New England J Med. (1999) 340:1169–75. 10.1056/NEJM199904153401505 10202168

[26] Cretolle C Fekete CN Jan D Nassogne MC Saudubray JM Brunelle F. Partial elective pancreatectomy is curative in focal form of permanent hyperinsulinemic hypoglycaemia in infancy: a report of 45 cases from 1983 to 2000. J Pediatr Surg. (2002) 37:155–8. 10.1053/jpsu.2002.30241 11819190

[27] Barthlen W Varol E Empting S Wieland I Zenker M Mohnike W. Surgery in focal congenital hyperinsulinism (CHI) - The “Hyperinsulinism Germany International” Experience in 30 Children. Pediatr. Endocrinol. Rev. (2016) 14:129–37. 10.17458/PER.2016.BVE 28508606

[28] Barthlen W. Surgery in congenital hyperinsulinism-tips and tricks not only for surgeons. A practical guide. Semin Pediatr Surg. (2011) 20:56–9. 10.1053/j.sempedsurg.2010.10.002 21186007

[29] Kapoor RR Flanagan SE Arya VB Shield JP Ellard S Hussain K. Clinical and molecular characterisation of 300 patients with congenital hyperinsulinism. Eur J Endocrinol. (2013) 168:557–64. 10.1530/EJE-12-0673 23345197 PMC3599069

[30] McAndrew HF Smith V Spitz L. Surgical complications of pancreatectomy for persistent hyperinsulinaemic hypoglycaemia of infancy. J Pediatr Surg. (2003) 38:13–6. 10.1053/jpsu.2003.50001 12592610

[31] Jack MM Greer RM Thomsett MJ Walker RM Bell JR Choong C. The outcome in Australian children with hyperinsulinism of infancy: early extensive surgery in severe cases lowers risk of diabetes. Clin Endocrinol. (2003) 58:355–64. 10.1046/j.1365-2265.2003.01725.x 12608942

[32] Beltrand J Caquard M Arnoux JB Laborde K Velho G Verkarre V. Glucose metabolism in 105 children and adolescents after pancreatectomy for congenital hyperinsulinism. Diabetes Care (2012) 35:198–203. 10.2337/dc11-1296 22190679 PMC3263917

[33] Fekete CN de Lonlay P Jaubert F Rahier J Brunelle F Saudubray JM. The surgical management of congenital hyperinsulinemic hypoglycemia in infancy. J Pediatr Surg. (2004) 39:267–9. 10.1016/j.jpedsurg.2003.11.004 15017535

[34] Lord K Radcliffe J Gallagher PR Adzick NS Stanley CA De Leon DD. High risk of diabetes and neurobehavioral deficits in individuals with surgically treated hyperinsulinism. J Clin Endocrinol Metab. (2015) 100:4133–9. 10.1210/jc.2015-2539 26327482 PMC4702456

[35] Capito C Khen-Dunlop N Ribeiro MJ Brunelle F Aigrain Y Cretolle C. Value of ^18^F-fluoro-L-dopa PET in the preoperative localization of focal lesions in congenital hyperinsulinism. Radiology. (2009) 253:216–22. 10.1148/radiol.2532081445 19709999

[36] Arnoux JB Verkarre V Saint-Martin C Montravers F Brassier A Valayannopoulos V. Congenital hyperinsulinism: current trends in diagnosis and therapy. Orphanet J Rare Dis. (2011) 6:63. 10.1186/1750-1172-6-63 21967988 PMC3199232

[37] Banerjee I Skae M Flanagan SE Rigby L Patel L Didi M. The contribution of rapid KATP channel gene mutation analysis to the clinical management of children with congenital hyperinsulinism. Eur J Endocrinol. (2011) 164:733–40. 10.1530/EJE-10-1136 21378087

[38] Snider KE Becker S Boyajian L Shyng SL MacMullen C Hughes N. Genotype and phenotype correlations in 417 children with congenital hyperinsulinism. J Clin Endocrinol Metab. (2013) 98:E355–63. 10.1210/jc.2012-2169 23275527 PMC3565119

[39] Treglia G Mirk P Giordano A Rufini V. Diagnostic performance of fluorine-18-dihydroxyphenylalanine positron emission tomography in diagnosing and localizing the focal form of congenital hyperinsulinism: a meta-analysis. Pediatr Radiol. (2012) 42:1372–9. 10.1007/s00247-012-2459-2 22885604

[40] Blomberg BA Codreanu I Cheng G Werner TJ Alavi A. Beta-cell imaging: call for evidence-based and scientific approach. Mol Imaging Biol. (2013) 15:123–30. 10.1007/s11307-013-0620-4 23413090

[41] Christiansen CD Petersen H Nielsen AL Detlefsen S Brusgaard K Rasmussen L. ^18^F-DOPA PET/CT and 68Ga-DOTANOC PET/CT scans as diagnostic tools in focal congenital hyperinsulinism: a blinded evaluation. Eur J Nucl Med Mol Imaging (2018) 45:250–61. 10.1007/s00259-017-3867-1 29116340 PMC5745571

[42] Banerjee I Forsythe L Skae M Avatapalle HB Rigby L Bowden LE. Feeding problems are persistent in children with severe congenital hyperinsulinism. Front Endocrinol. (2016) 7:8. 10.3389/fendo.2016.00008 26903946 PMC4747152

[43] Takano S Ito Y Watanabe Y Yokoyama T Kubota N Iwai S. Pancreaticojejunostomy versus pancreaticogastrostomy in reconstruction following pancreaticoduodenectomy. Br J Surg. (2000) 87:423–7. 10.1046/j.1365-2168.2000.01395.x 10759736

[44] Peranteau WH Bathaii SM Pawel B Hardy O Alavi A Stanley CA. Multiple ectopic lesions of focal islet adenomatosis identified by positron emission tomography scan in an infant with congenital hyperinsulinism. J Pediatr Surg. (2007) 42:188–92. 10.1016/j.jpedsurg.2006.09.046 17208563

[45] Meissner T Wendel U Burgard P Schaetzle S Mayatepek E. Long-term follow-up of 114 patients with congenital hyperinsulinism. Eur J Endocrinol. (2003) 149:43–51. 10.1530/eje.0.1490043 12824865

